# A Novel Three-Dimensional and Tissue Doppler Echocardiographic Index for Diagnosing and Prognosticating Heart Failure With Preserved Ejection Fraction

**DOI:** 10.3389/fcvm.2022.822314

**Published:** 2022-02-10

**Authors:** Weiding Wang, Guanyu Mu, Changle Liu, Juan Xie, Hao Zhang, Xiaowei Zhang, Jingjin Che, Gary Tse, Tong Liu, Guangping Li, Huaying Fu

**Affiliations:** ^1^Tianjin Key Laboratory of Ionic-Molecular Function of Cardiovascular Disease, Department of Cardiology, Tianjin Institute of Cardiology, The Second Hospital of Tianjin Medical University, Tianjin, China; ^2^School of Public Health, Tianjin Medical University, Tianjin, China; ^3^Faculty of Health and Medical Sciences, University of Surrey, Guildford, United Kingdom; ^4^Kent and Medway Medical School, Canterbury, United Kingdom

**Keywords:** heart failure with preserved ejection fraction, diagnosis, prognosis, a novel echocardiography index, right heart catheterization

## Abstract

**Introduction:**

The diagnosis of heart failure with preserved ejection fraction (HFpEF) remains challenging. In this study, a novel echocardiography index based on three-dimensional and tissue Doppler echocardiography for diagnosing and estimating prognosis in HFpEF.

**Materials and Methods:**

Patients with symptoms and/or signs of heart failure and normal left ventricular ejection fraction (LVEF ≥50%) who underwent right heart catheterization were screened. Patients were divided based on pulmonary capillary wedge pressure (PCWP) of ≥15 mmHg and PCWP <15 mmHg. A diagnosis of HFpEF was confirmed by PCWP of ≥15 mmHg according to ESC guidelines. A novel index was calculated by the ratio between stroke volume standardized to body surface area (SVI) and tissue Doppler mitral annulus systolic peak velocity S' (SVI/S'). Its diagnostic and prognostic values were determined.

**Results:**

A total of 104 patients (mean age 64 ± 12 years) were included. Of these, 63 had PCWP ≥15 mmHg and 41 patients had PCWP <15 mmHg. Compared to the PCWP <15 mmHg group, the ≥15 mmHg group had a significantly lower SVI/S' (*P* < 0.001). Logistic regression showed that SVI/S' was associated with high PCWP measured invasively. The SVI/S' had an area under the curve of 0.761 for diagnosing classifying between PCWP ≥15 mmHg and <15 mmHg. Kaplan–Meier analysis showed that the lower SVI/S' group showed a poorer prognosis.

**Conclusions:**

SVI/S' is a non-invasive index calculated by three-dimensional and tissue Doppler echocardiography. It is a surrogate measure of PCWP and can be used to diagnose and determine prognosis in HFpEF.

## Introduction

Heart failure (HF) with preserved ejection fraction (HFpEF), previously known as diastolic heart failure, is a complex clinical syndrome, characterized by normal or near normal left ventricular ejection fraction (LVEF > 50%) (1). HFpEF results from abnormalities of ventricular relaxation and reduced ventricular compliance can lead to impairment of effective filling in the left ventricle (LV), in turn lead to declines in stroke volume (SV) and thus cardiac output (2). Community-based cohort studies have found that HFpEF accounts for ~40–50% of incident HF cases (3, 4). The diagnosis of HFpEF requires clinical symptoms and/or signs of HF, as well as evidence of preserved LVEF and diastolic dysfunction (1). Echocardiography has emerged as the most commonly used and widely available diagnostic tool in patients with suspected HFpEF (5). A wide variety of echocardiography parameters have been used to assess diastolic function, including mitral valve inflow velocities (E), mitral annular velocity (e'), ratio of early diastolic mitral valve inflow velocity and early diastolic mitral annulus tissue velocity (E/e'), peak velocity of tricuspid regurgitation (TR) jet and left atrial maximum volume index (LAVI) (1, 6). However, the sensitivity and specificity of the echocardiography parameters for diagnosing HFpEF remains unsatisfactory (7). As a result, the assessment of invasive hemodynamic using cardiac catheterization continues to serve as the gold standard for diagnosing diastolic dysfunction (8).

In this study, proposed a novel echocardiography index based on three-dimensional echocardiography, calculated by stroke volume standardized by the body surface area (SVI) and tissue Doppler mitral annulus systolic peak velocity (S'), SVI/S'. The diagnostic and prognostic value of this SVI/S' in HFpEF was investigated.

## Materials and Methods

### Study Population

This is a prospective study. This study was registered in the Chinese Clinical Trial Registry network (registration number: ChiCTR1900024903). The study fully conformed to the ethical guidelines laid down by the 1975 Declaration of Helsinki. The study protocol was approved by the ethics committee of the second hospital of Tianjin Medical University (2019, 010). All patients provided written informed consent.

Consecutive patients who underwent cardiac catheterization with an initial diagnosis of HFpEF at the Second Hospital of Tianjin Medical University between December 2019 and June 2021 were screened. The exclusion criteria are congenital heart disease, restrictive cardiomyopathy, constrictive pericarditis, chronic obstructive pulmonary disease, pulmonary embolism, idiopathic pulmonary hypertension, cardiac surgery history and valvular disease (valve stenosis, moderate or severe regurgitation). All enrolled patients were confirmed by cardiologists at admission and underwent a detailed physical examination and laboratory testing including hematologic and biochemical variables and NT-proBNP. The initial diagnosis of HFpEF requires clinical symptoms and/or signs of HF and normal ejection fraction (LVEF ≥50%) by subsequent three-dimensional transthoracic echocardiography (3D-TTE).

### Definitions of Signs and Symptoms Suggestive of Heart Failure

Typical symptoms included dyspnea, orthopnea, paroxysmal nocturnal dyspnea, reduced exercise tolerance, fatigue, tiredness, increased time to recover after exercise and/or ankle swelling. The specific signs for heart failure included elevated jugular venous pressure, hepatojugular reflux, third heart sound (gallop rhythm) and laterally displaced apical impulse.

### Transthoracic Echocardiography

Echocardiography was performed and reported by cardiologists with advanced training in echocardiography. Images were obtained in the left recumbent position of the patients. The measurements were based on current guidelines for the assessment of cardiovascular structure and function (9). Transthoracic echocardiography was performed using a Philips IE33 ultrasound system equipped with a X5-1 probe. Images with at least five cycles of sinus rhythm and atrial fibrillation (AF) (echocardiography measurements require at least five cycles in patients with AF) were digitally stored in the original DICOM format for offline analysis. The images were recorded in the following standard views: parasternal long-axis view, parasternal short-axis view, apical four-chamber view, apical three-chamber view and apical two-chamber view. The following parameters were determined: left ventricular end-diastolic diameter (LVEDD), left ventricular wall thickness, LVEF, left ventricular end-diastolic volume (LVEDV) and end-systolic volume (LVESV), tissue Doppler mitral annulus (septal side) systolic peak velocity (S'), left ventricular global longitudinal strain (GLS), the ratio of early diastolic transmitral velocity to early diastolic mitral annular tissue velocity (E/e'), the ratio of early diastolic transmitral velocity to diastolic transmitral velocity (E/A), left atrial diameter (LAD), left atrial volume index (LAVI), myocardial performance index (Tei index) and tricuspid annular plane systolic excursion (TAPSE). HFA-PEFF scores (10) were calculated after echocardiography examination.

Three-dimensional full-volume scan was obtained from the apical position and ensured including the whole LV structure in the 3D full-volume image. LVEF, LVEDV, LVESV, left ventricular stroke volume (LVSV) and left atrial volume (LAV) were measured with 3D Auto mode (11). LVSV and LAV were standardized to body surface area (BSA) (SVI and LAVI, respectively). The mean values of three measurements of the SVI/S' were then calculated.

### Right Heart Catheterization

All patients had a clinical indication for right heart catheterization (RHC) based on heart failure guidelines (1). RHC were performed within 24 hours after echocardiography examination in all the patients. A 6F balloon-tipped Swan-Ganz catheter was inserted into the median cubital vein for venous access by Seldinger technique. A two-chamber Swan-Ganz thermosensitive floating catheter was placed through the sheath and sent to the superior vena cava, the right atrium, the right ventricle, the main pulmonary artery and the right lower pulmonary artery, then recorded the pressure at the above points. After the Swan-Ganz floating catheter was sent to the distal end of the pulmonary artery, the balloon was pulled up and wedged into the pulmonary arterioles, and the mean pulmonary capillary wedge pressure (PCWP) was measured. Catheterization data were acquired through automated measurements on typical pressure waveforms.

### Stratification

The study was carried out using single blinding. According to the 2016 ESC guidelines for heart failure, PCWP ≥15 mmHg was recommended as the gold standard for HFpEF diagnosis (1). Based on the results of right heart catheterization, the patients were divided into two groups: HFpEF group (PCWP ≥15 mmHg) and non-HFpEF group (PCWP <15 mmHg).

### Primary Outcome and Statistical Analysis

The primary outcome was a composite event of cardiovascular death and re-hospitalization due to heart failure within 6 months of discharge. Statistical analyses were performed by using SPSS 26 and MedCalc 19 software. Continuous variables were expressed as mean ± SD or medians (25th−75th percentiles) and categorical variables as numbers and percentages. Tests for significance were conducted using the unpaired *t*-test or non-parametric test (Mann–Whitney *U* test) for continuous variables and the Chi-square test or Fisher's exact test for categorical variables. Correlation analysis was conducted using variables that were statistically significant (*P* < 0.05). To identify factors that were relevant to high PCWP (≥15 mmHg), logistic regression analysis was then conducted with echocardiography parameters and NT-proBNP as the independent variables and the presence/absence of high PCWP as the dependent variable. The optimal cut-off value of SVI/S' for diagnosis was investigated using a receiver-operating characteristics (ROC) analysis. The optimum cut-off value was defined as the point combining the highest sensitivity and specificity. The event-free survival rate was estimated by the Kaplan–Meier method with group comparisons made using the log-rank test. Statistical tests were performed at 95% confidence intervals and a *P*-value ≤0.05 was considered statistically significant. Coefficient of variation from duplicate measurements was used to compare stroke volumes and S' inter- or intro-observers.

## Results

### Baseline Characteristics and Laboratory Measurements

**Figure 1:**
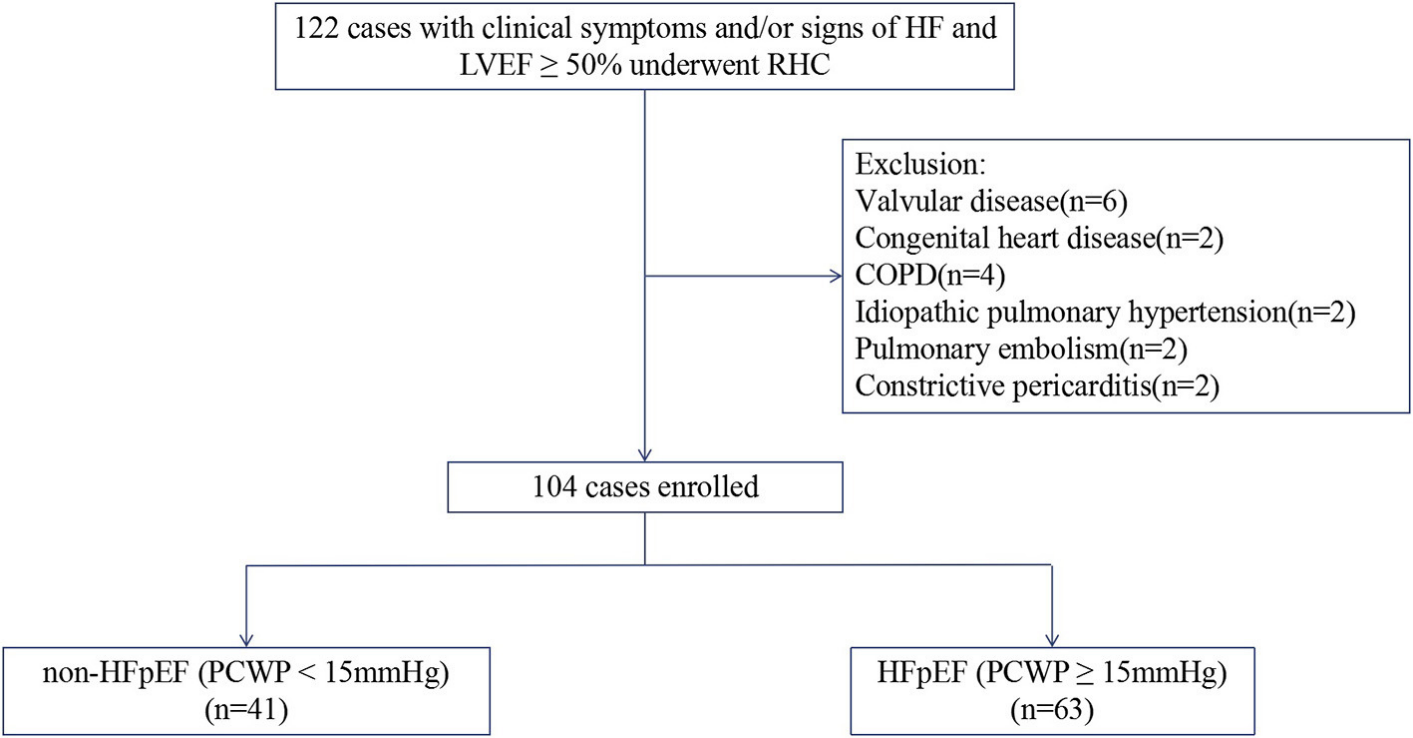
Flowchart of study selection. HF, heart failure; LVEF, left ventricular ejection fraction; RHC, right heart catheterization; COPD, chronic obstructive pulmonary disease; HFpEF, Heart failure with preserved ejection fraction; PCWP, pulmonary capillary wedge pressure.

The study flowchart for subject inclusion and exclusion is shown in Figure 1. 122 patients with typical symptoms and/or signs of heart failure and normal ejection fraction (LVEF ≥50%) underwent right heart catheterization examination were screened for this study. Following the exclusion of 18 patients, 104 patients were finally included. The study cohort had a mean age of 64 ± 12 years, 57% were male, with a mean LVEF of 60 ± 7% and mean PCWP of 18 ± 8 mmHg. 63 (61%) patients were identified as having increased PCWP (≥15 mmHg; HFpEF group), with the remaining 41 (39%) patients showing normal PCWP (<15 mmHg; non-HFpEF group).

The baseline characteristics of the two groups are summarized in Tables 1, 2. HFpEF group showed higher BMI and higher rates of coronary heart disease (CHD) and atrial fibrillation (AF). There were no significant differences in age, gender, body surface area (BSA), blood pressure, smoking, alcohol intake, medications, the prevalence of hypertension and diabetic history. Higher levels of NT-proBNP and percent of patients with HFA-PEFF score in 5-6 points were observed in HFpEF group. There was no significant difference in percent of patients with HFA-PEFF score in 2–4 points between the two groups. There were no significant differences in hepatic and renal function, red blood cell distribution levels and serum lipids between the two groups.

**Table 1:** Clinical characteristics of patients.

**Parameter**	**Non-HFpEF (*n* = 41)**	**HFpEF (*n* = 63)**	***P***
Baseline characteristic			
Age (years)	64 ± 13	65 ± 12	0.879
Male, *n* (%)	18 (42.3)	36 (52.4)	0.266
BMI (kg/m^2^)	25.4 ± 3.9	26.9 ± 4.0	0.029^*^
BSA (m^2^)	1.8 ± 0.2	1.9 ± 0.2	0.102
HR (bpm)	75 ± 13	74 ± 14	0.884
SBP (mmHg)	137 ± 24	136 ± 21	0.965
DBP (mmHg)	83 ± 14	81 ± 11	0.642
Medical history			
Hypertension, *n* (%)	34 (83.8)	48 (76.2)	0.378
CHD, *n* (%)	20 (48.7)	44 (69.8)	0.038^*^
AF, *n* (%)	4 (9.7)	18 (28.5)	0.025^*^
DM, *n* (%)	6 (14.6)	17 (26.9)	0.159
Smoke, *n* (%)	8 (19.5)	15 (23.8)	0.605
Alcohol intake, *n* (%)	3 (7.3)	10 (15.8)	0.195
Medications			
ARB, *n* (%)	14 (34.1)	30 (47.6)	0.213
ACEI, *n* (%)	4 (9.7)	12 (19.0)	0.223
Beta-blocker, *n* (%)	21 (51.2)	36 (57.1)	0.679
CCB, *n* (%)	14 (34.1)	30 (47.6)	0.213
Diuretic, *n* (%)	12 (29.2)	14 (22.2)	0.350
Anti-platelet, *n* (%)	23 (56.1)	43 (68.3)	0.270
HFA-PEFF score, median (IQR)	2 (1–2)	3 (2–4)	<0.001^*^
0-1, *n* (%)	14 (34.1)	0 (0)	<0.001^*^
2-4, *n* (%)	26 (63.4)	49 (77.7)	0.146
5-6, *n* (%)	1 (2.4)	14 (22.2)	0.009^*^

*ACEI, angiotensin converting enzyme inhibitor; AF, atrial fibrillation; ARB, angiotensin receptor blocker; BMI, body mass index; CCB, calcium channel blocker; CHD, coronary heart disease; DBP, diastolic blood pressure; DM, diabetes mellitus; HR, heart rate; IQR, interquartile range; SBP, systolic blood pressure*.

* *P < 0.05*.

**Table 2:** Laboratory parameters of patients.

**Parameter**	**Non-HFpEF (*n* = 26)**	**HFpEF (*n* = 42)**	***P***
WBC (^*^10^9^/L)	6.8 ± 1.5	7.2 ± 2.7	0.414
*N* (%)	68.5 ± 9.1	65.9 ± 8.2	0.155
RDW-CV	13.2 ± 1.2	13.3 ± 1.5	0.786
RDW-SD	42.7 ± 3.6	42.2 ± 6.1	0.600
Hb (g/L)	135 ± 20	132 ± 29	0.689
Plt (^*^10^9^/L)	237 ± 55	215 ± 72	0.113
ALT (IU/L)	20 ± 15	22 ± 18	0.493
AST (IU/L)	18 ± 6	21 ± 12	0.248
Cr (umol/L)	89 ± 42	87 ± 36	0.779
Ur (mmol/L)	7.0 ± 2.5	6.9 ± 2.7	0.900
K^+^ (mmol/L)	4.2 ± 0.4	4.0 ± 0.5	0.109
Na^+^ (mmol/L)	142 ± 2	141 ± 3	0.244
TG (mmol/L)	1.9 ± 1.8	1.8 ± 1.2	0.881
TC (mmol/L)	4.9 ± 1.0	4.5 ± 1.1	0.110
HDLc (mmol/L)	1.2 ± 0.4	1.1 ± 0.3	0.133
LDLc (mmol/L)	3.1 ± 0.8	2.8 ± 0.8	0.099
Glu (mmol/L)	6.5 ± 2.7	6.4 ± 2.2	0.807
CK (U/L)	87 ± 51	103 ± 66	0.224
CKMB (U/L)	13 ± 10	14 ± 7	0.403
NT-proBNP (pg/ml)	154 ± 111	231 ± 171	0.016^*^

*ALT, alanine aminotransferase; AST, aspartic transaminase; CK, creatine kinase; CKMB, creatine kinase isoenzyme; Cr, creatinine; Glu, glucose; Hb, hemoglobin; HDLc, high density lipoprotein cholesterol; LDLc, low density lipoprotein cholesterol; N, percent of neutrophile granulocyte; NT-proBNP, N-terminal pro brain natriuretic peptide; PCWP, pulmonary capillary wedge pressure; Plt, platelet; RDW-CV, red blood cell distribution width-variable coefficient; RDW-SD, red blood cell distribution width-standard deviation; TC, total cholesterol; TG, triglyceride; Ur, urea; WBC, white blood cell*.

* *P < 0.05*.

### Baseline Echocardiography and Right Heart Catheterization

Compared to patients in the non-HFpEF group, those in the HFpEF group demonstrated higher LAD, IVST, PWT, E/e' and LAVI, and lower LVEDD, LVEF, LVSV and lower SVI/S'. By contrast, no significant differences in left ventricular volume, blood flow velocity, S', Tei index, GLS and TAPSE between the two groups were observed (Table 3). HFpEF patients had higher PCWP and pulmonary artery systolic pressure (PASP) in RHC examinations.

**Table 3:** Echocardiographic and invasive parameters of patients.

**Parameter**	**Non-HFpEF (*n* = 26)**	**HFpEF (*n* = 42)**	***P***
LAD (mm)	38.8 ± 6.0	42.4 ± 6.8	0.013^*^
IVST (mm)	9.2 ± 1.9	10.1 ± 2.2	0.039^*^
PWT (mm)	9.0 ± 1.4	9.8 ± 1.9	0.040^*^
LVEDD (mm)	48 ± 2	45 ± 4	0.039^*^
LVEDV (ml)	84 ± 17	77 ± 20	0.126
LVESV (ml)	30 ± 9	32 ± 8	0.470
LVSV (ml)	53 ± 14	46 ± 13	0.012^*^
LVEF (%)	63 ± 7	58 ± 7	0.001^*^
E/A	0.9 ± 0.3	0.8 ± 0.3	0.456
E/e'	11.6 ± 3.4	14.0 ± 5.0	0.014^*^
S' (cm/s)	6.8 ± 1.5	7.2 ± 1.2	0.271
TEI	0.36 ± 0.14	0.43 ± 0.12	0.180
GLS (%)	−18.5 ± 4.0	−15.9 ± 4.4	0.152
TAPSE (mm)	17.5 ± 2.9	16.9 ± 2.6	0.327
LAVI (ml/m^2^)	25.9 ± 7.9	29.5 ± 8.3	0.047^*^
SVI/S' [(ml/m^2^)/(cm/s)]	4.4 ± 1.2	3.4 ± 1.0	<0.001^*^
PASP (mmHg)	21 ± 8	29 ± 7	0.009^*^
PCWP (mmHg)	11 ± 3	22 ± 8	<0.001^*^

*E/A, the ratio of early diastolic transmitral velocity to diastolic transmitral velocity; E/e', the ratio of early diastolic transmitral velocity to early diastolic septal myocardial velocity; GLS, left ventricular global longitudinal strain rate; LAD, left atrium diameter; LAVI, left atrial volume index; LVEDD, left ventricular end-diastolic diameter; LVEDV, left ventricular end-diastolic volume; LVEF, left ventricular ejection fraction; LVESV, left ventricular end-systolic volume; LVSV, left ventricular stroke volume; IVST, interventricular septal thickness; PASP, pulmonary artery systolic pressure; PCWP, pulmonary capillary wedge pressure; PWT, posterior wall thickness; SVI, the ratio of body surface area standardized stroke volume; SVI/S', the ratio of body surface area standardized stroke volume (SVI) normalized by tissue Doppler mitral annulus systolic peak velocity; TAPSE, tricuspid annular plane systolic excursion*.

* *P < 0.05*.

### Logistic Regression and ROC Curve Analysis

There was a poor to moderate correlation between echocardiography parameters, NT-proBNP, HFA-PEFF score and PCWP (Table 4). Logistic regression analysis, E/e' and SVI/S' remained associated with invasively measured high PCWP (≥15 mmHg) (OR = 1.2 *P* = 0.037; OR = 0.3 *P* = 0.005, respectively).

**Table 4:** Correlations between parameters and PCWP.

**Parameter**	***r***	***p***
LAD (mm)	0.300	0.004^*^
IVST (mm)	0.257	0.015^*^
PWT (mm)	0.276	0.009^*^
LVEDD (mm)	0.253	0.232
LVSV (ml)	−0.215	0.039^*^
LVEF (%)	−0.187	0.075
E/e'	0.492	0.001^*^
LAVI (ml/m^2^)	0.556	0.001^*^
SVI/S' [(ml/m^2^)/(cm/s)]	−0.431	0.013^*^
NT-proBNP (pg/ml)	0.473	0.001^*^
HFA-PEFF score	0.552	<0.001^*^

*E/e', the ratio of early diastolic transmitral velocity to early diastolic septal myocardial velocity; LAD, left atrium diameter; IVST, interventricular septal thickness; LAVI, left atrial volume index; LVEDD, left ventricular end-diastolic diameter; LVEF, left ventricular ejection fraction; LVSV, left ventricular stroke volume; NT-proBNP, N-terminal pro brain natriuretic peptide; PCWP, pulmonary capillary wedge pressure; PWT, posterior wall thickness; SVI/S', the ratio of body surface area standardized stroke volume (SVI) normalized by tissue Doppler mitral annulus systolic peak velocity; TAPSE, tricuspid annular plane systolic excursion*.

* *P < 0.05*.

**Figure 2:**
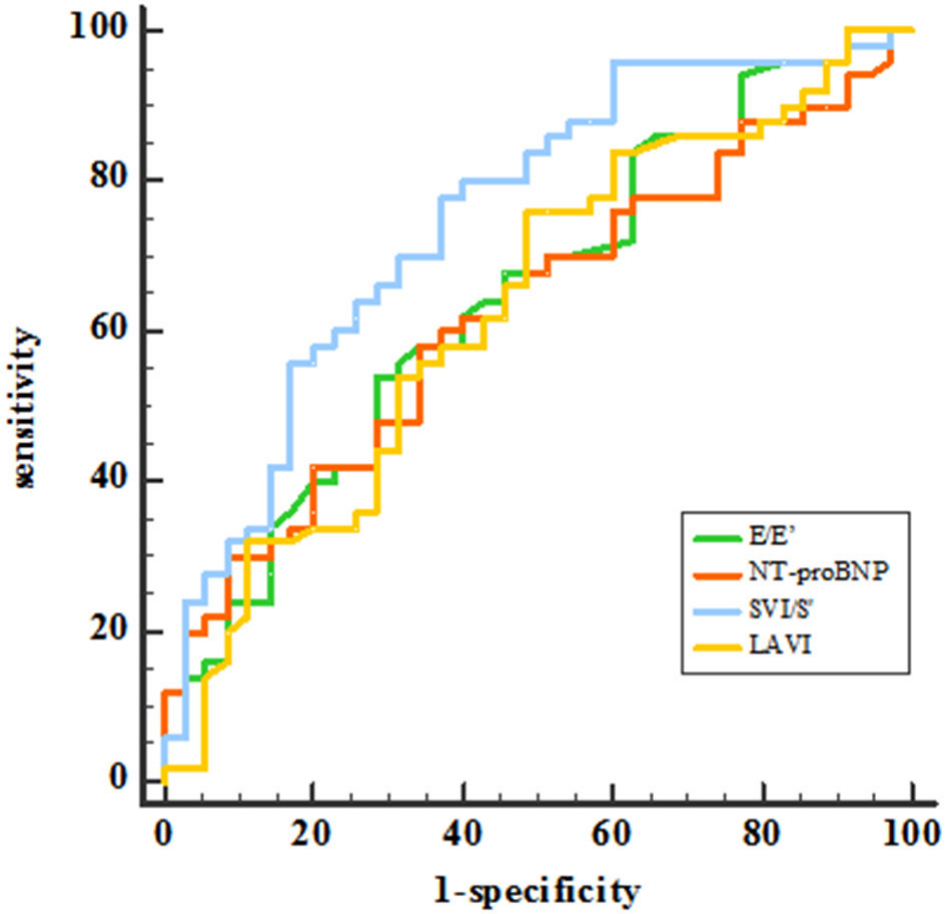
ROC analysis showed the SVI/S', E/e', LAVI and NT-proBNP diagnosing ability. SVI/S', the ratio of body surface area standardized stroke volume (SVI) and tissue Doppler mitral annulus systolic peak velocity; E/e', the ratio of early diastolic transmitral velocity to early diastolic septal myocardial velocity; LAVI, left atrial volume index; NT-proBNP, N-terminal pro-brain natriuretic peptide.

Receiver operating characteristic (ROC) analysis was applied using a PCWP cut-off of ≥15 mmHg. This yielded an AUC for SVI/S' of 0.761 (*P* < 0.001). The cut-off value of SVI/S' was 4.08. The SVI/S' cut-off value was considered for diagnosing HFpEF with a sensitivity of 80% and a specificity of 64%. Figure 2 and Table 5 showed the ability of SVI/S', E/e', LAVI and NT-proBNP to distinguish HFpEF from non-HFpEF. The AUC of these parameters for diagnosing HFpEF were 0.761, 0.646, 0.638, and 0.630 respectively.

**Table 5:** Receiver-operating characteristic curves for the diagnosis of HFpEF.

**Parameter**	**Sensitivity**	**Specificity**	**AUC**	**95%CI**	***P***
SVI/S'	80%	65%	0.761	0.660–0.844	<0.001^*^
E/e'	53%	71%	0.646	0.540–0.739	0.012^*^
LAVI	77%	50%	0.638	0.529–0.737	0.023^*^
NT-proBNP	59%	64%	0.630	0.524–0.727	0.025^*^

*AUC, area under the curve; CI, confidence interval; E/e', the ratio of early diastolic transmitral velocity to early diastolic septal myocardial velocity; LAVI, left atrial volume index; NT-proBNP, N-terminal pro-brain natriuretic peptide; SVI/S', the ratio of body surface area standardized stroke volume (SVI) normalized by tissue Doppler mitral annulus systolic peak velocity*.

* *P < 0.05*.

There was a correlation between the HFA–PEFF score and SVI/S' (*r* = −0.326, *P* = 0.002). Compared with the non-HFpEF group, lower SVI/S' was observed in the HFpEF group (4.3 ± 1.1 vs. 3.3 ± 0.7, *P* < 0.001) in the patients with intermediate HFA–PEFF score (2–4 points). With the cut-off value of 4.08, the positive and negative predictive value of SVI/S' for diagnosing HFpEF in the patients with an intermediate HFA–PEFF score (2–4 points) were 75 and 78%, respectively.

### Follow-Up

**Figure 3:**
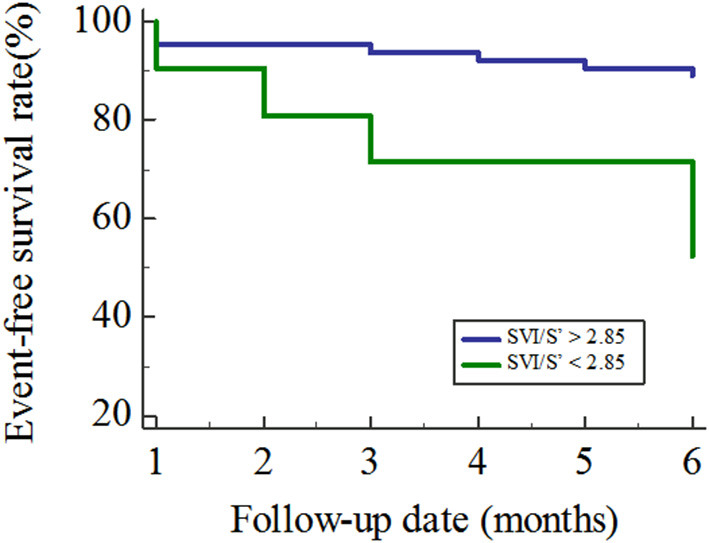
Kaplan–Meier curves for the freedom from the primary outcome. SVI/S', the ratio of body surface area standardized stroke volume (SVI) and tissue Doppler mitral annulus systolic peak velocity.

A total of 84 patients finished the following up in this study, and 21 patients met the endpoints during the follow-up periods. All 21 patients were re-hospitalized due to worsening of heart failure and no patient died from cardiovascular events. By ROC analysis, the best cut-off value of SVI/S' for predicting poor outcome was <2.85 (AUC = 0.688, *P* = 0.021). Kaplan–Meier analysis showed that patients with SVI/S' <2.85 had a poorer prognosis than those with SVI/S'>2.85 (*P* = 0.001, Figure 3).

### Feasibility and Reproducibility

Measurements of SVI/S' could be performed in all cases. 20 patients were randomly selected from all, and the data of them were evaluated by two independent cardiologists. Intra- and inter-observer variability for SV and S' was 4.71, 6.54, 4.20, and 5.69%, respectively. SV and S' were measured again by one observer to verify the inter-observer agreement after an interval of 1 month.

## Discussion

The main findings of this study are that the novel non-invasive echocardiographic index, stroke volume standardized to body surface area (SVI) divided by tissue Doppler mitral annulus systolic peak velocity S' (SVI/S'), can be used for both diagnosis and determining prognosis in HFpEF.

In recent years, HFpEF has received a considerable amount of attention from the medical community (12, 13), but its diagnosis and prognosis remain difficult (1, 14). Patients with HFpEF are more often women, obese and more commonly have a history of hypertension and AF (1). According to current ESC guidelines, the diagnosis of HFpEF requires signs and/or symptoms of heart failure, a preserved ejection fraction and evidence of structural and/or functional cardiac abnormalities, indicating diastolic dysfunction. Assessment of diastolic function can provide valuable insight into the development and progression of HFpEF, allowing for earlier detection and intervention (15). At present, the gold standard is to determine intracardiac pressures with invasive hemodynamic measurements using right and left heart catheterization (1). This method provides a wealth of information and parameters for measuring diastolic function, which is central to the definition of diastolic dysfunction (DD) and HFpEF. In the present study, PCWP assessed by RHC was selected as the gold standard for diagnosing HFpEF. However, heart catheterization is an invasive and time-consuming procedure, which is not appropriate as a universal approach to all patients with suspected HFpEF (16). As a result, echocardiography has served as the most commonly used method owing to its wide availability and non-invasive nature (17). A wide variety of echocardiography techniques and parameters have been used to establish a diagnosis of heart failure and determine the EF sub-type, which is recommended by the guide and widely accepted in clinical practice, including E/e' ratio, peak velocity of TR jet and LAVI.

However, significant limitations of these echocardiographic parameters for the assessment of diastolic function and diagnosis of HFpEF are present. In patients with HFpEF, echocardiographic measurements have a poor predictive value for the estimation of invasively acquired left ventricular end-diastolic pressure (LVEDP) and PCWP. This limitation should be taken into account for the diagnosis and evaluation of patients with HFpEF. The E/e' ratio is generally accepted for estimating increased left ventricular filling pressures and has received a prominent position in current guidelines and recommendations (18). The E/e' ratio was validated in several studies against invasively measured mean left ventricular diastolic pressure, PCWP and LVEDP across a variety of populations and etiologies with varying results (9, 15). The correlation between E/e' and PCWP in our study was moderate (*r* = 0.470), and in bivariate logistic regression analysis, it was independently associated with invasively measured high PCWP, which is consistent with previously published results. Different results were found in other traditional echocardiographic parameters, including LAVI, TAPSE, and NT-proBNP. Although specific, current recommendations show low sensitivity, identifying only 34–60% of subjects with invasively proven HFpEF based on these traditional echocardiographic data alone (16). LVEF is normal in patients with HFpEF, but myocardial deformation observed by speckle-tracking echocardiography (STE) often shows abnormalities. A recent study employing STE found that systolic function measures such as LV global longitudinal strain (GLS) are frequently abnormal in HFpEF patients (19). This study shows similar results, but the impaired GLS brings out no significance compared with non-HFpEF patients. In the present study, while HFpEF has a higher NT-proBNP level and the correlation between NT-proBNP and PCWP is moderate, NT-proBNP shows no diagnostic ability from ROC curve analysis.

In 2019, the Heart Failure Association of the European Society of Echocardiography recommended a new stepwise diagnostic process, the “HFA–PEFF diagnostic algorithm” (10). The algorithm contains a diagnostic scoring system, which has functional, morphological, and biomarker domains, including echocardiographic measurements of cardiac structure and function and NP levels. Within each domain, a major criterion scores 2 points or a minor criterion 1 point. A total score ≥5 points is considered to be diagnostic of HFpEF, while a score of ≤1 point is considered to make a diagnosis of HFpEF very unlikely and to mandate investigations for alternative causes. Patients with an intermediate score (2–4 points) need further functional tests, such as exercise stress echocardiography or invasive hemodynamic tests. In the present study, scores were significantly different between the non-HFpEF group and HFpEF group. There was a moderate correlation between the HFA–PEFF score and PCWP. Moreover, higher HFA-PEFF scores (≥5 points) had high positive predictive value to diagnose HFpEF (93%). Previous findings also substantiate the diagnostic validity of the HFA-PEFF scoring systems for the identification of HFpEF patients (20, 21). Nevertheless, the score leaves us with a rather large group (~72%) of patients with an intermediate likelihood where additional testing is required, which is consistent with recently published results (20–22).

### Novel Echocardiographic Parameter, SVI/S'

After the determination of the LVEF, evaluation of diastolic function is crucial for the workup of HFpEF. Diastolic dysfunction is defined as impaired myocyte relaxation or increased wall stiffness, resulting in decreased filling and elevated pressures during diastole (23), and further a reduced stroke volume. Recent studies have demonstrated the decreasing of SV measured by invasive hemodynamic or echocardiography method, despite the preserved left ventricular ejection fraction in HFpEF patients (24, 25). SV, which can be affected by contractility, preload and afterload, is lower in not only systolic dysfunction but also diastolic dysfunction. This is different from EF being reduced only when systolic dysfunction occurs. SVI, stroke volume standardized by BSA, can accurately reflect the change of SV in HFpEF patients. S', a tissue Doppler parameter, which refers to the mitral annulus systolic peak velocity during contraction, directly reflects the left ventricular systolic function. As mentioned above, left ventricular systolic function in HFpEF patients is generally normal, even increased (26), so we use the ratio of these two parameters to improve the sensitivity of diagnosis.

**Figure 4:**
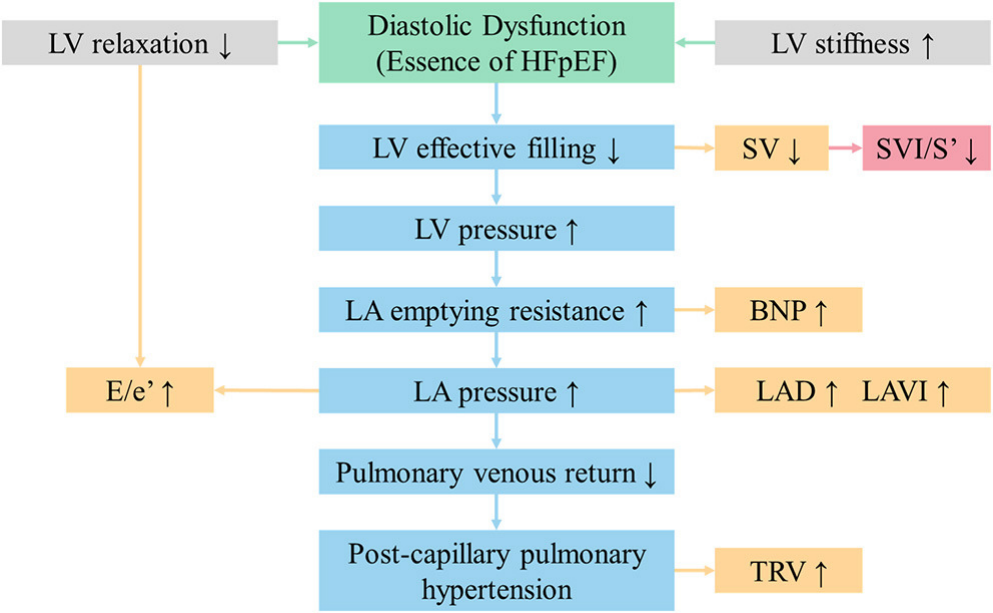
SVI/S' is the initial changing echocardiographic parameter in the process of HFpEF. BNP, brain natriuretic peptide; E/e', the ratio of early diastolic transmitral velocity to early diastolic septal myocardial velocity; HFpEF, heart failure with preserved ejection fraction; LA, left atrium; LAD, left atrial diameter; LAVI, left atrial volume index; LV, left ventricular; SVI/S', the ratio of body surface area standardized stroke volume (SVI) and tissue Doppler mitral annulus systolic peak velocity; TRV, tricuspid regurgitation velocity.

As shown in Figure 4, a lack of effective filling in LV due to diastolic dysfunction may result in the following results. Firstly, the insufficient filling of LV leads to reductions in SV and consequently cardiac output (2). Secondly, an increase in LV stiffness, reflecting abnormal diffuse myocardial fibrosis, will produce an increase in E/e' and left ventricular mass index. Thirdly, the increased pressure in the LV chamber can lead to increased pressure in LA, followed by a gradual enlargement in LAV and LAVI (23). Meanwhile, the poor pulmonary venous return may bring about post-capillary pulmonary hypertension, which is characterized by greater tricuspid regurgitation and pressure gradient. In summary, decreasing SV should be the initial change in the process of HFpEF. SVI/S' decreasing was more sensitive and occurred earlier than other indexes.

Therefore, we propose this echocardiographic parameter and try to explore the value of SVI/S' in identifying HFpEF. A lower SVI/S' was previously found in HFpEF patients and SVI/S' showed better diagnostic value than traditional parameters. Better correlations between SVI/S' and PCWP (*r* = −0.435) were found, and SVI/S' remained independently associated with invasively measured high PCWP by logistic regression after multivariable adjustment. Meanwhile, ROC analysis resulted in an AUC for SVI/S' of 0.761 (*P* < 0.001), in contrast with E/e' and LAVI (yielded 0.694 and 0.614, respectively). Notably, NT-proBNP showed poor diagnostic ability from ROC curves (*P* = 0.578, *P* = 0.169) in the present study. Indeed, the evidence cited in the guidelines on the diagnostic utility of NT-proBNP derives from patients with predominantly systolic dysfunction (1). Thus, even though the guidelines recommended, the diagnostic utility of NT-proBNP in HFpEF patients is still conflicting (27). Further analysis suggested a correlation between the HFA–PEFF score and SVI/S'. Moreover, SVI/S' may be valuable in distinguishing HFpEF in patients with intermediate HFA–PEFF score. Thus, the combination of the two methods may provide a more accurate diagnosis in future research.

At present, the 2018 Chinese guideline for diagnosis and treatment of Heart failure suggests that natriuretic peptide levels can be used to evaluate the prognosis of HFpEF, but the value is still controversial. Several studies show that natriuretic peptide is mainly related to cardiac systolic function, but there is no effective evidence that natriuretic peptide is associated with the diastolic function (28). In addition to natriuretic peptide, C-reactive protein, galactose lectin-3 and left atrial enlargement can be used to predict the prognosis of HFpEF, but the predictive accuracy is not clear (29–31). In this study, the ROC curve of SVI/S' for predicting the occurrence of HFpEF endpoint events showed an AUC of 0.688, indicating that SVI/S' could independently predict the poor outcome. The Kaplan–Meier analysis directly showed that the risk of events in patients with SVI/S' <2.85 was significantly higher than that in patients with SVI/S' >2.85 (*P* = 0.001), indicating that SVI/S' <2.85 was a predictor of poor prognosis.

Studies have shown that 3D echocardiography of adequate quality can improve the quantification of LV volumes and LVEF and has the best accuracy compared with values obtained through CMR (32). With the more accessible 2D echocardiography, the SV is usually obtained from the product of the left ventricular outflow tract (LVOT) cross-sectional area (CSA, in cm^2^) with the LVOT velocity–time integral (VTI) (33). The LVOT CSA is derived from the LVOT diameter (LVOTd) using the formula π*r*^2^ [3.1416 × (LVOTd/2)^2^], or its equivalent (LVOTd)^2^ × 0.785. For the LVOT diameter, using a similar inner edge-to-inner edge methodology, the measurement should be made ~3–10 mm from the valve plane in midsystole (34, 35). The LVOT VTI is obtained by tracing the envelope of the Doppler spectrum of LVOT systolic flow from the apical five- or three-chamber view using pulsed-wave Doppler (PWD), with the sample volume placed within the LVOT, approximately at 5 mm distance to the aortic valve (11). The SV calculated with 2D mode may be more pragmatic and useful in the daily work.

## Limitations

Some limitations of this study should be acknowledged. Firstly, our study was performed in a single-center, and the size of the study population was relatively small. Secondly, right heart catheterizations and echocardiography were not performed simultaneously may have a potential impact on the results. Moreover, S' was calculated on the septal side instead of the mean of the lateral and the septal side tissue Doppler velocity. Finally, right heart catheterizations were performed in rest and stressed RHC data were not available.

## Conclusions

SVI/S' is a non-invasive index calculated by three-dimensional and tissue Doppler echocardiography. It is a surrogate measure of PCWP and can be used to diagnose and determine prognosis in HFpEF.

## Data Availability Statement

The raw data supporting the conclusions of this article will be made available by the authors, without undue reservation.

## Ethics Statement

The study protocol was approved by the Ethics Committee of the Second Hospital of Tianjin Medical University (2019, 010). The patients/participants provided their written informed consent to participate in this study.

## Author Contributions

HF and JX conceived and designed the study. WW, GM, CL, HZ, XZ, TL, and JC worked on the acquisition of the data. WW and GM analyzed the data and drafted the manuscript. GT, GL, and HF revised the manuscript critically. All authors contributed to the article and approved the submitted version.

## Funding

The study was supported by grants to HF from Tianjin Natural Science Foundation (Nos. 16JCYBJC25000 and 17JCYBJC27800), Key Laboratory Scientific Research Foundation of Second Hospital of Tianjin Medical University (Nos. 2018ZDSYS03 and 2019ZDSYS03), and Clinical study of Second Hospital of Tianjin Medical University (No. 2019LC03). The study was funded by Tianjin Key Medical Discipline (Specialty) Construction Project.

## Conflict of Interest

The authors declare that the research was conducted in the absence of any commercial or financial relationships that could be construed as a potential conflict of interest.

## Publisher's Note

All claims expressed in this article are solely those of the authors and do not necessarily represent those of their affiliated organizations, or those of the publisher, the editors and the reviewers. Any product that may be evaluated in this article, or claim that may be made by its manufacturer, is not guaranteed or endorsed by the publisher.
